# Molecular Identification of Endophytic Bacteria in *Leucojum aestivum* In Vitro Culture, NMR-Based Metabolomics Study and LC-MS Analysis Leading to Potential Amaryllidaceae Alkaloid Production

**DOI:** 10.3390/ijms22041773

**Published:** 2021-02-10

**Authors:** Rosella Spina, Sahar Saliba, François Dupire, Agata Ptak, Alain Hehn, Séverine Piutti, Sophie Poinsignon, Sebastien Leclerc, Sabine Bouguet-Bonnet, Dominique Laurain-Mattar

**Affiliations:** 1 Université de Lorraine, CNRS, L2CM, F-54000 Nancy, France; sahar.saliba@ssc-dietetique.fr (S.S.); francois.dupire@univ-lorraine.fr (F.D.); 2 Department of Plant Breeding, Physiology and Seed Science, University of Agriculture in Krakow, Łobzowska 24, 31-140 Krakow, Poland; mfptak@cyf-kr.edu.pl; 3 Université de Lorraine, INRAE, LAE, F-54000 Nancy, France; alain.hehn@univ-lorraine.fr (A.H.); severine.piutti@univ-lorraine.fr (S.P.); 4 Université de Lorraine, CNRS, CRM2, F-54000 Nancy, France; sophie.poinsignon@univ-lorraine.fr (S.P.); sabine.bonnet@univ-lorraine.fr (S.B.-B.); 5 Université de Lorraine, CNRS, LEMTA, F-54000 Nancy, France; sebastien.leclerc@univ-lorraine.fr

**Keywords:** *Leucojum aestivum*, in vitro culture, *Bacillus* sp., endophytic bacteria, metabolomics, Nuclear Magnetic Resonance, Mass Spectrometry

## Abstract

In this study, endophytic bacteria belonging to the *Bacillus* genus were isolated from in vitro bulblets of *Leucojum aestivum* and their ability to produce Amaryllidaceae alkaloids was studied. Proton Nuclear Magnetic Resonance (^1^H NMR)-based metabolomics combined with multivariate data analysis was chosen to compare the metabolism of this plant (in vivo bulbs, in vitro bulblets) with those of the endophytic bacteria community. Primary metabolites were quantified by quantitative ^1^H NMR (qNMR) method. The results showed that tyrosine, one precursor of the Amaryllidaceae alkaloid biosynthesis pathway, was higher in endophytic extract compared to plant extract. In total, 22 compounds were identified including five molecules common to plant and endophyte extracts (tyrosine, isoleucine, valine, fatty acids and tyramine). In addition, endophytic extracts were analyzed using Liquid Chromatography-Mass Spectrometry (LC-MS) and Gas Chromatography-Mass Spectrometry (GC-MS) for the identification of compounds in very low concentrations. Five Amaryllidaceae alkaloids were detected in the extracts of endophytic bacteria. Lycorine, previously detected by ^1^H NMR, was confirmed with LC-MS analysis. Tazettine, pseudolycorine, acetylpseudolycorine, 1,2-dihydro-chlidanthine were also identified by LC-MS using the positive ionization mode or by GC-MS. In addition, 11 primary metabolites were identified in the endophytic extracts such as tyramine, which was obtained by decarboxylation of tyrosine. Thus, *Bacillus* sp. isolated from *L. aestivum* bulblets synthesized some primary and specialized metabolites in common with the *L.* *aestivum* plant. These endophytic bacteria are an interesting new approach for producing the Amaryllidaceae alkaloid such as lycorine.

## 1. Introduction

Amaryllidaceae plants produce a huge arsenal of chemical compounds known as related isoquinoline alkaloids, which include the well-known galanthamine, lycorine and tazettine. These specialized metabolites show very complex structures and possess various biological activities.

Galanthamine is used for the treatment of Alzheimer’s disease [1]. Lycorine possesses the ability to inhibit in vitro replication of the poliomyelitis virus, herpes simplex virus, Bunyamwera virus, West Nile virus, dengue virus and Severe Acute Respiratory Syndrome-Coronavirus (SARS-CoV) [2,3,4,5]. This alkaloid also exhibits antimalarial [6] and cytotoxic activities, which have led to clinical trials to determine the use of lycorine in the treatment of cancer [7].

In the last decade, several studies made evidence that a plant has to be considered as a holobiont, comprising the host plant and its associated microbiota. Every living plant on Earth is host to one or more endophytes: bacteria or fungi that colonize living plant tissue producing a wide variety of specialized metabolites without causing disease [8]. However, their biodiversity within the plant kingdom has not yet been assessed. Likewise, information concerning plant–endophyte interactions is nonexistent or poorly understood.

Several endophytic microbes present in plants were also described to be able to synthesize such biomolecules alone or in close interaction with plants, [9,10]. It has been recently reported in our group that the *Curvularia papendorfii* endophytic fungus can produce both known and novel structures [11]. Some strains of endophytes could potentially modulate the level of production of metabolites of interest in the model plant. A complex molecular dialogue between endophytic microorganisms and plants can modulate the growth and tolerance of plants to stress, which will lead to the increased production of a large diversity of specialized metabolites.

Concerning the Amaryllidaceae plants, only few studies have reported the isolation of endophytic fungi and bacteria from plants of this family: *Lycoris radiata* [12], *Crinum macowanii* [13,14] and *Narcissus tazetta* [15]. Some endophytes isolated from *L. radiata* are able to promote the concentration of alkaloids and plant growth [12]; however comprehensive studies about their metabolic capacity has not been conducted.

Currently, to study the metabolism of plants and microorganisms, the main approaches rely on Nuclear Magnetic Resonance (NMR) and Mass Spectrometry (MS) [16,17,18,19,20,21]. In the literature, Amaryllidaceae plants are investigated using metabolomic approaches. A metabolomic analysis by Gas Chromatography-Mass Spectrometry (GC-MS) was developed for the determination of Amaryllidaceae alkaloids, sugar and amino acids and quantification of galanthamine in *Leucojum aestivum* and *Narcissus* ssp. The authors have used unsupervised multivariate data analysis technique to separate different species [22].

A metabolomic analysis by Ultra Performance Liquid Chromatography-Quadrupole Time-of-Flight Mass Spectrometry(UPLC-QTOF-MS) was realized using the bulbs, roots, leaves and stems of *Narcissus pseudonarcissus* ‘King Alfred’ to present a profile of Amaryllidaceae alkaloids and create a metabolome database [23].

Some examples in Amaryllidaceae plants using ^1^H NMR metabolomics studies are the authentication of *Narcissus pseudonarcissus* in in vivo bulbs [24,25]; the metabolic profiles of alkaloids in bulbs, leaves and roots depending of seasonal variations [26]; the evaluation of alkaloid variation related to the age of *Narcissus* bulbs [27]; and the profile of Amaryllidaceae alkaloids in the tissue culture of *Narcissus pseudonarcissus* cv. Carlton [28].

*Leucojum aestivum* L. plant synthesizes Amaryllidaceae alkaloids such as galanthamine and lycorine. Different biotechnological approaches were reported in the literature with a view to producing these alkaloids with a high added value [29].

A clear correlation between the accumulation of these alkaloids and cell differentiation as has been previously reported concerning the induction of bulblets with auxins [30]) and the elicitation of biosynthesis Amaryllidaceae alkaloids in *L. aestivum* L. plants cultured with carbohydrates in a temporary immersion system (RITA^®^ bioreactor, Vitropic, Saint-Mathieu-de-Tréviers, France) has been described [31].

However, the quantities of alkaloids produced in tissue cultures remain insufficient to consider large-scale production. Endophytic bacteria or fungi can synthesize specialized metabolites like their host plants and are considered to be an interesting source of biomolecules [8]. In addition, in plant tissue cultures like *Echinacea* [32], or in some genotypes of the *Juglans* species [33] endophytic bacteria have been identified. These endophytic bacteria communities often act on plant growth promoting effectors [34,35].

In our study, for the first time, *Leucojum aestivum* bulblets induced in in vitro conditions were investigated to discover the capacity of the endophytic bacteria community to synthesize specialized metabolites.

NMR-based metabolomics was chosen as the method for comparing the metabolism of *L. aestivum* in vivo bulbs, in vitro bulblets and the isolated endophyte obtained from the in vitro bulblet. The ^1^H NMR-based metabolomics method with multivariate analysis provides a metabolomic fingerprint in the biological samples, which describes both qualitatively and quantitatively the metabolites present in a living organism at a precise moment. This is especially true of the *L. aestivum* in vivo bulb and in vitro bulblet and isolated endophytic bacteria, so that the specific metabolic signatures and the quantification of identified metabolites can be established. The ability of endophyte bacterium isolate from in vitro bulblets of *L. aestivum* to produce Amaryllidaceae alkaloids was investigated. The enriched extract of isolated endophyte, obtained after Solid-Phase Extraction (SPE) was extensively studied by NMR, LC-MS and GC-MS analysis. To our knowledge, the investigation of the presence of Amaryllidaceae alkaloids and the identification of specialized metabolites in the endophyte from bulblets of *L. aestivum* has never been realized.

## 2. Results and Discussion

### 2.1. Isolation and Identification of Endophytic Bacteria from Leucojum aestivum In Vitro Bulblets

**Figure 1:**
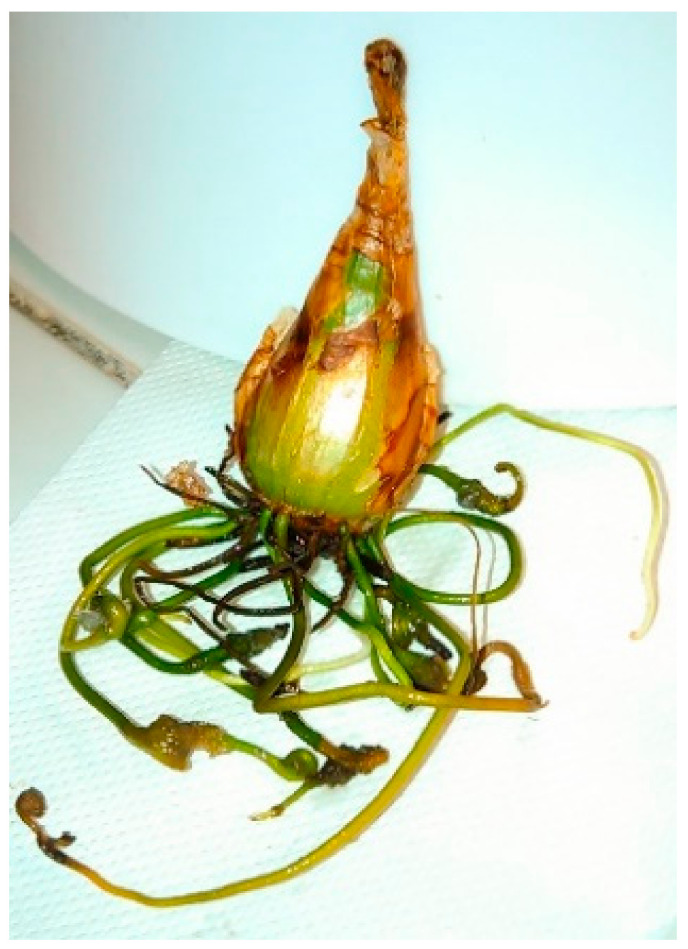
*L. aestivum* in vitro bulblet.

The potential presence of bacterial endophytes was investigated in in vitro bulblets of *L. aestivum* (Figure 1). This research had never been described before.

Of the 300 explants obtained from bulblets, 20 bacterial isolates and four endophytic bacteria were determined to be morphologically unique. These bacteria were designated as endophytes because they are microorganisms that do not visibly harm the host but live in the inner parts of the plants and can be isolated from in vitro bulblets. It is important to note that the bulblets were never in contact with the microorganisms of the rhizosphere; however, they were regenerated after several propagation cycles from primary explants isolated from the leaves inside in vivo bulbs. The endophytic microorganisms survive in microplantlets undetected [36], and their identification is important since they may have positive, neutral or negative impacts on their host plants [35]. The Gram stain reaction indicated that they are Gram-positive bacilli. Polymerase Chain reaction (PCR) analyses, targeted at 16S Ribonucleic acid (RNA) sequences, was carried out on the four bacteria strains.

**Figure 2:**
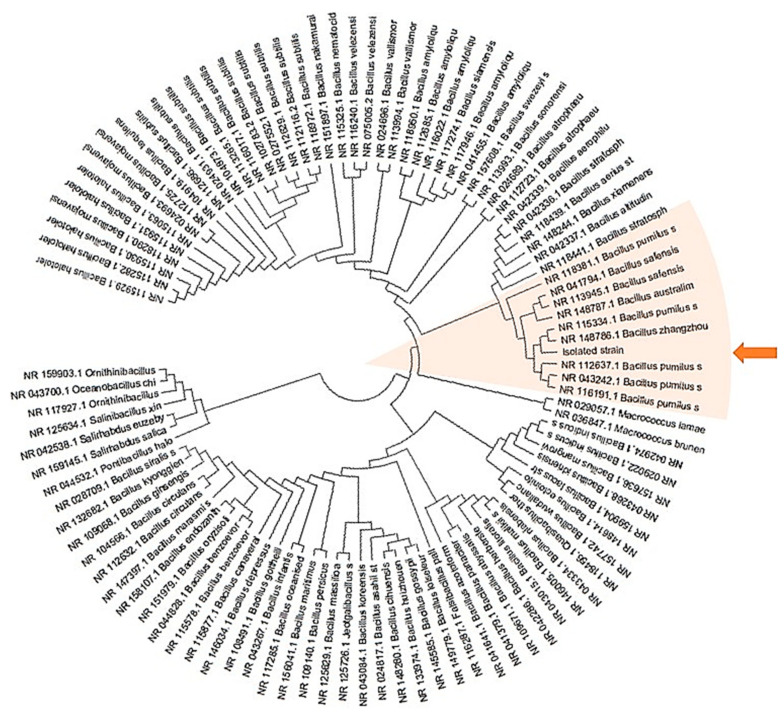
Evolutionary analysis by Maximum Likelihood method and identification of *Bacillus* genus.

The results obtained indicate 100% identity of the four isolates with the targeted sequence. A search for sequence similarity carried out on a RNA16S database shows that the four isolates belong to the *Bacillus* genus and are closest to the *Bacillus safensis*, *pumilus* and *zhangzhouensis* species (Figure 2, orange arrow).

The isolated strain (highlighted by an orange arrow) has been compared to all bacteria presented in the Figure 2 and described in the insertion S1 in supplementary materials. The tree with the highest log likelihood (−13485.60) is shown. Initial tree(s) for the heuristic search were obtained automatically by applying Neighbor-Join and BioNJ algorithms to a matrix of pairwise distances estimated using the Maximum Composite Likelihood (MCL) approach, and then selecting the topology with superior log likelihood value. This analysis involved 101 nucleotide sequences. There were a total of 1600 positions in the final dataset.

*Bacillus* spp. are frequently isolated from seeds and plant tissues. Antagonistic activities against pathogenic fungi are described for different strains belonging to many species of *Bacillus* notably *B. pumilus* and *B. subtilis. B. pumilus* is described as a biocontrol agent and commercialized for these properties by a life sciences company [37]. *B. pumilus* is also an endophytic bacteria isolated from different species of *Echinacea* [32] and *Citrus* plants [38]. Only the genus *Bacillus* was isolated in in vitro bulblets of *L. aestivum* as compared with endophytic bacteria that were isolated from the Amaryllidaceae bulbs of *Crinum macowanii* of which three to seven bacterial genera were identified [13,14]. In *Lycoris radiata* 30 bacterial genera were identified [12]. These various results could be associated with the selected plant tissue [39].

### 2.2. Untargeted ^1^H NMR-Based Metabolomics: Identification and Quantification

**Figure 3:**
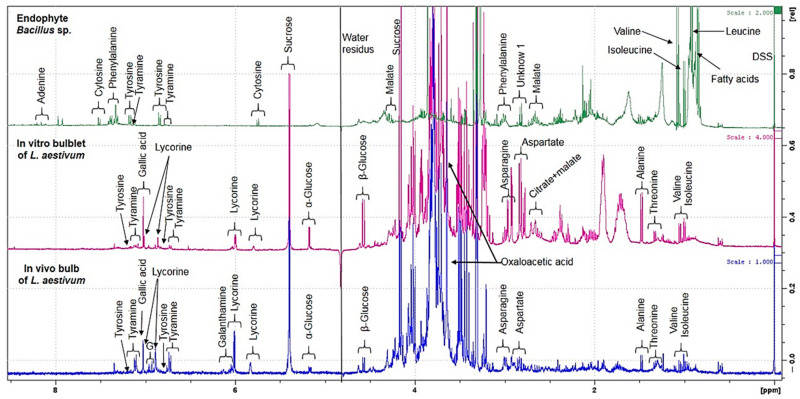
Selected ^1^H NMR spectra of methanol–water extracts of in vivo bulb and in vitro bulblet of *L. aestivum* and endophyte *Bacillus* sp. All analyses were performed in triplicate.

The ^1^H NMR spectra of the three biological samples––in vivo bulb and in vitro bulblets of *L. aestivum* and *Bacillus* sp. endophyte isolated from in vitro bulblets––were performed. The representative ^1^H NMR spectra of methanol–water extracts, are shown in Figure 3.

By untargeted ^1^H NMR-based metabolomics, it was shown that all the extracts have in common four primary metabolites (tyrosine, isoleucine, valine, fatty acids) and a monoamine, tyramine.

An inspection of the proton spectra of *L. aestivum* in vivo bulbs revealed the presence of seven amino acids: tyrosine, asparagine, aspartate, alanine, threonine, valine and isoleucine. In addition, the signals of phenylethylamine such as tyramine and organic acids such as gallic acid and oxaloacetic acid were observed. Regarding Amaryllidaceae alkaloids, the characteristic signals of both lycorine and galanthamine were identified in the in vivo bulb. These results were confirmed by comparison of original standards recorded in the same conditions of analysis.

An inspection of the proton spectra of the *L. aestivum* in vitro bulblets, revealed a similar spectrum profile to that of the in vivo bulbs, except for the presence of citrate and malate, which were only observed in the tissue cultures. However, the characteristic signals of galanthamine were not present in the in vitro bulblets while lycorine was observed.

In both in vitro bulblets and in vivo bulbs, the sugars sucrose, α-glucose and β-glucose were present. It is known that sugars are involved in the expression of genes implicated in both primary and specialized metabolisms [40].

The presence of tyrosine and tyramine was emphasized in the in vivo bulbs [26] and in different tissue cultures obtained from *Narcissus pseudonarcissus* [28].

In the extract of *Bacillus* sp. endophyte, the ^1^H NMR spectrum revealed the presence of six amino acids: phenylalanine, tyrosine, alanine, isoleucine, valine and leucine. The characteristic signals of a nitrogenous base, adenosine and cytosine, are visible. In the endophyte *Bacillus* sp. extract, it is interesting to note the presence of tyramine, which can be synthesized by decarboxylation of tyrosine via the enzyme tyrosine decarboxylase. Tyrosine and tyramine are two of the biosynthetic precursors of Amaryllidaceae alkaloids and tyramine is one starting point in the biosynthesis pathway [41].

Using ^1^H NMR-based metabolomic analysis it was possible to identify in a single step 22 metabolites with differences in polarity, chemical structure, stability and concentration. The detailed information for the assigned peaks can be found in Table 1.

### 2.3. Statistical Analysis with Multivariate Data Analysis

**Figure 4:**
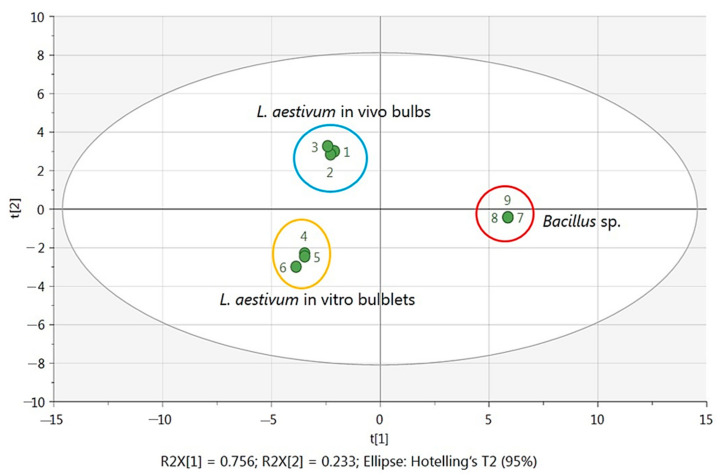
PCA score plot of ^1^H NMR spectra from *Bacillus* sp. endophyte, in vivo bulbs and in vitro bulblets of *L. aestivum*.

A Principal Components Analysis (PCA) was chosen to represent the obtained data and to explore the correlation of the samples. The PCA score plot (Figure 4) revealed that the first and second principal axis accounted for 75.6% and 23.3% of the total variability, respectively.

The PCA ordination clearly discriminated the biological extracts according to their biological origin (*L. aestivum* in vivo bulbs, in vitro bulblets and *Bacillus* sp.) with weak variability between the three replicates of each origin.

**Figure 5:**
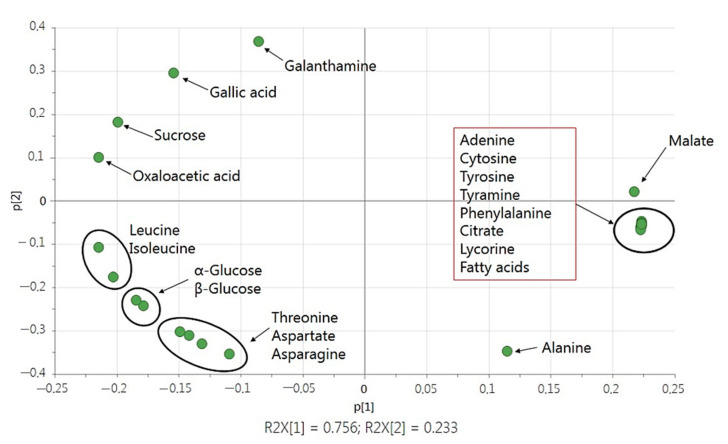
PCA loading plot of ^1^H NMR spectra of the variables from *Bacillus* sp. endophyte, in vivo bulbs and in vitro bulblets of *L. aestivum*.

The loading plot shows the contribution of the respective metabolites to the discrimination. The correlation circle shows on plane 1–2 that the metabolites adenine, cytosine, tyrosine, tyramine, phenylalanine, citrate, fatty acids and lycorine form a separate group. It was opposed to the group represented by the variables galanthamine, gallic acid, sucrose and oxaloacetic acid. The other three groups are represented by the pair leucine and isoleucine, α-glucose and β-glucose and the three amino acids threonine, aspartate and asparagine. From these identified signals, it was clear that adenine, cytosine, tyrosine, tyramine, phenylalanine, citrate as well as lycorine and fatty acids were more predominant in *Bacillus* sp. Endophytes were located on the positive side of PC1. Meanwhile, metabolites located on the negative side of PC1 were identified exclusively in plant extracts of *L. aestivum*. Metabolites that discriminated more in in vitro bulblets were leucine, isoleucine, α-glucose, β-glucose, threonine, aspartate and asparagine while galanthamine, gallic acid, sucrose and oxaloacetic acid were more discriminated in in vivo bulbs (Figure 5).

### 2.4. Quantification by NMR

**Figure 6:**
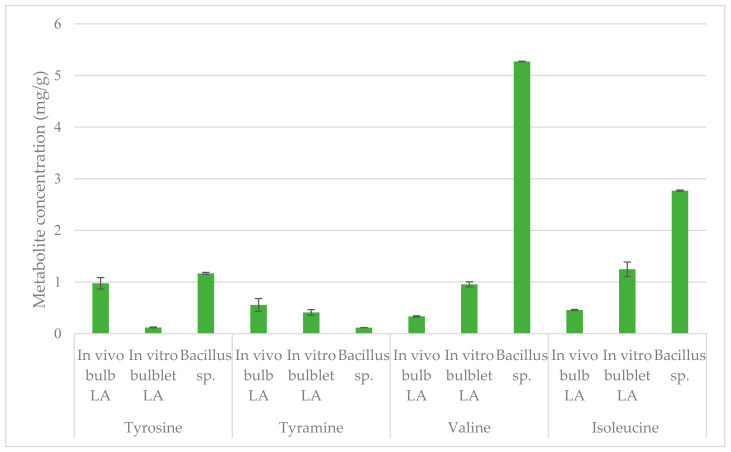
Metabolites quantified by qNMR present in in vivo bulb, in vitro bulblet of *L*. *aestivum* (LA) and endophyte *Bacillus* sp. extracts. Data are given as mean ± SD, replicates are *n* = 3, *p* < 0.01. The concentration is expressed as mg/g DW.

In this study 10 metabolites (tyrosine, tyramine, valine, isoleucine, threonine, alanine, α-glucose, β-glucose, sucrose and lycorine) were quantified using relative NMR quantification (qNMR). Three amino acids (tyrosine, valine and isoleucine) and the monoamine tyramine (Figure 6) were quantified in in vivo bulbs, in vitro bulblets of *L. aestivum* and endophytic extracts. The characteristic signal of tyrosine (6.84 ppm), tyramine (6.72 ppm), valine (1.05 ppm), and isoleucine (0.96 ppm), were chosen for the quantification.

Higher concentrations of two amino acids are observed, 5.27 mg/g of dry weight (DW) for valine and 2.77 mg/g of DW for isoleucine, in endophyte *Bacillus* extract comparatively to the others amino acids in the different extracts tested. Interestingly, the concentration of tyrosine was higher in endophytic extract with 1.17 mg/g of DW by comparison to in vivo extract (0.97 mg/g of DW) and in vitro extract (0.12 mg/g of DW). However, the amount of the decarboxylated compound, tyramine, present in the *Bacillus* sp. extract (0.12 mg/g of DW) was the lowest comparatively to the others extracts.

**Figure 7:**
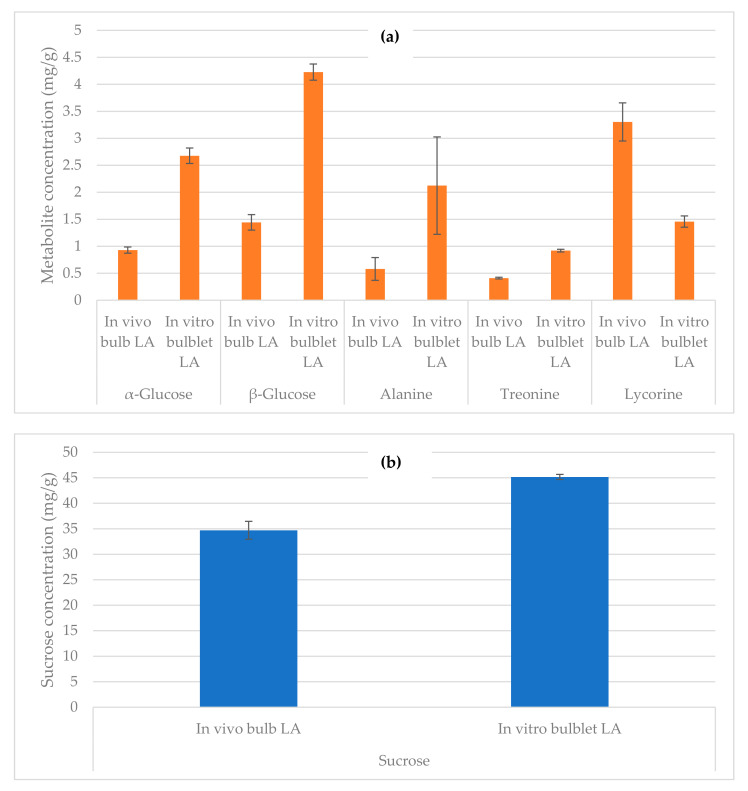
Metabolites quantified by qNMR present in in vivo bulbs and in vitro bulblets of *L. aestivum* (LA). (**a**) α-glucose, β-glucose, alanine, threonine and lycorine contents expressed as mg/g of dry weight (DW); (**b**) sucrose contents expressed as mg/g DW. Data are given as mean ± SD, *p* < 0.01.

Other metabolites in in vivo bulbs and in vitro bulblets of *L. aestivum* extracts were quantified (Figure 7).

Three sugars (α-glucose, β-glucose and sucrose), two amino acids (threonine, alanine) and one Amaryllidaceae alkaloid (lycorine) were distinguished. The characteristic signal of α-glucose (5.17 ppm), β-glucose (4.57 ppm), sucrose (5.39 ppm), threonine (1.33 ppm), alanine (1.46 ppm) and lycorine (6.85 ppm), were chosen for the quantification.

In the in vivo bulb of *L. aestivum*, the concentration of α-glucose was 0.93 mg/g of DW and β-glucose of 1.44 mg/g of DW. In contrast, the highest amounts of α-glucose (2.67 mg/g of DW) and β-glucose (4.22 mg/g of DW) were observed in in vitro bulblets. Sucrose was the sugar most abundant in both tissues. The concentration of sucrose was 34.7 mg/g of DW and 45.2 mg/g of DW respectively in in vivo bulb and in vitro bulblets. Alanine was present in in vivo bulb with a concentration of 0.58 mg/g of DW and in vitro bulblets 2.12 mg/g of DW. The concentration of threonine was 0.41 mg/g of DW and 0.92 mg/g of DW in in vivo bulb and in in vitro bulblets respectively.

The amount of lycorine was 3.30 mg/g of DW in in vivo extract and 1.46 mg/g of DW in in vitro extract of *L. aestivum.*

The main source of lycorine is plant extraction, which is limited by the low concentration in Amaryllidaceae plants (varying from trace to 1.73 mg/g), seasonal variations [42] and depletions of wild Amaryllidaceae populations [43]. In vitro cultures offer an alternative approach for obtaining galanthamine and lycorine metabolites and several methods are described in a recent review [44]. By in vitro cultures the concentration of lycorine up to 1.13 mg/g of DW in *Leucojum aestivum* ‘Gravity Giant’ bulblets [29]. In *L. aestivum*, lycorine content is known to vary between in vivo bulbs and in vitro bulblets regenerated in various conditions of plant tissue cultures [29,45].

A negative correlation can be observed between the presence of lycorine and the presence of amino acids (alanine and threonine) and sugars (α-glucose and β-glucose). Also lycorine biosynthesis decreased by the addition of various concentrations of glucose in the culture medium of *L. aestivum* plants [31]. Using qNMR, in a single step of analysis, the quantification of the primary and specialized metabolites is realized and a global overview of our three biological samples (in vivo bulb, in vitro bulblets and isolated endophyte) has been presented.

### 2.5. Target Research of Amaryllidaceae Alkaloids by ^1^H NMR, LC-MS and GC-MS Analysis in Bacterial Endophyte Bacillus sp.

A selective purification for the research of Amarylidaceae alkaloids, by a solid-phase extraction (SPE) was used. The methanolic extract of bacterial endophyte *Bacillus* sp. was subjected to SPE for the removal of sugars and to concentrate the sample. The purified extract was evaporated. The dry residues were dissolved in deuterated methanol solvent. The enriched extract was analyzed by NMR using the same sequence used for the ^1^H NMR metabolomic analysis.

**Figure 8:**
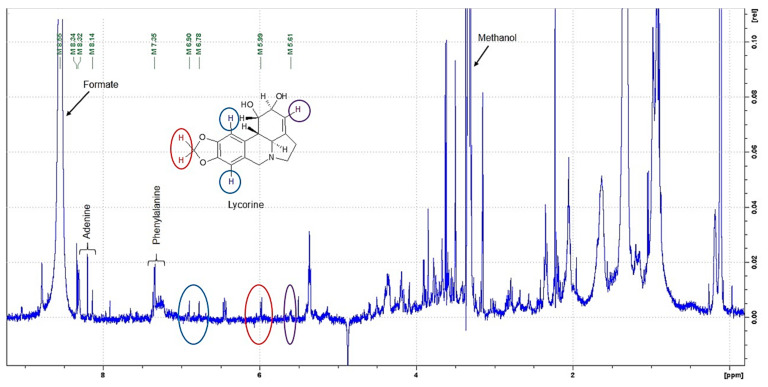
Representative one-dimensional ^1^H NMR spectrum of *Bacillus* sp. isolated from in vitro bulblets of *L. aestivum* after purification by solid-phase extraction (SPE) strong cation exchange (SCX) cartridge. The colors (blue, red and violet) display some characteristic signals of a lycorine structure.

Inspection of the ^1^H NMR spectrum (Figure 8) showed signals of a purine derivative, adenine, at 8.32 ppm, the aromatic signals at 7.35 ppm of phenylalanine and the characteristic signals of lycorine never described before. Clearly, the signals at 6.90 ppm and 6.78 ppm in blue, 5.99 ppm in red and 5.61 ppm in violet are in an uncrowded region without interference from other signals in the mixtures.

Liquid Chromatography coupled with high-resolution Mass Spectrometry (LC-MS) analysis was realized using a purified methanolic extract of endophytic bacteria *Bacillus* sp. using the same conditions of analysis as in our previous works. The bulbs and bulblets of *L. aestivum* were extensively investigated by LC-MS to show the presence of Amaryllidaceae alkaloids [29].

LC-MS profiling generated an extensive mass list corresponding to a wide variety of metabolites in the endophytic extract of *Bacillus* sp. from the *L. aestivum* in vitro bulblets. The LC-MS analysis performed in positive-ion mode confirmed the presence of lycorine previously detected by ^1^H NMR analysis. Additional information was obtained such as the detection of eight compounds including four Amaryllidaceae alkaloids (lycorine, tazettine, pseudolycorine and acetyl pseudolycorine) (Table 2), and four other molecules (methyl-phenylalanine, deoxyadenosine, adenosine, deoxycytidine). These results were confirmed using different databases such as GNPS [46], Metlin [47], Massbank [48] and Sirius [49]. Also a house-made data base (DataNat database, n° IDDN: FR.001.480019.000.S.P.2020.000.10300) was used to confirm the chemical identification.

Lycorine was confirmed in the endophytic extract by the analysis of the mass spectrum (C_16_H_17_NO_4_, [M + H]^+^ 288.1230) and the retention time (10.1 ± 0.2 min). The same result was obtained for commercial standard lycorine (Supplementary Figure S1).

The alkaloid tazettine (C_18_H_21_NO_4_ [M + H]^+^ 332.1486) was identified in bulbs of different varieties of *Narcissus tazetta* [50], *Galanthus nivalis* [51] but not in *L. aestivum*. However, tazettine was detected in this endophytic extract of *Bacillus* and in plant tissue culture of *L. aestivum* [31]. In the literature, bacterial endophytes are recognized as a rich and diverse source of alkaloids [52] such as camptothecin [53]. In general, *Bacillus* spp. are the most abundant metabolite-producing bacteria endophytes [54]. *Bacillus* endophytes have been reported to produce aromatic compounds, polysaccharides and plant hormones, thus representing higher potential in crop management strategies [55]. Endophytic *Bacillus* sp. was able to induce the highest level of diosgenin biosynthesis in *Trigonella foenum-graecum* [39].

The GC-MS analysis was also used to investigate the chemical composition of purified extract from endophytic bacteria. The results are shown in the Table 3. Two compounds showed MS fragmentation patterns characteristic of Amaryllidaceae alkaloids, specifically tazettine and 1,2-dihydro-chlidanthine. The alkaloids were identified by comparing the measured data with published data [46] and using the NIST library [56]. Tazettine and 1,2-dihydro-chlidanthine were identified in *N. tazetta* [50].

To resume our investigation, for the first time the metabolic screening showed the presence of Amaryllidaceae alkaloids in endophytic *Bacillus* sp. isolated from in vitro bulblets of *L. aestivum*. The endophyte was able to synthesize lycorine and its derivatives from an ortho-para’ phenol oxidative coupling of precursor 4′-*O*-methyl-norbelladine, tazettine from a para-para’ phenol oxidative coupling and 1,2-dihydro-chlidanthine from a para-ortho’ phenol oxidative coupling of this precursor [57].

**Figure 9:**
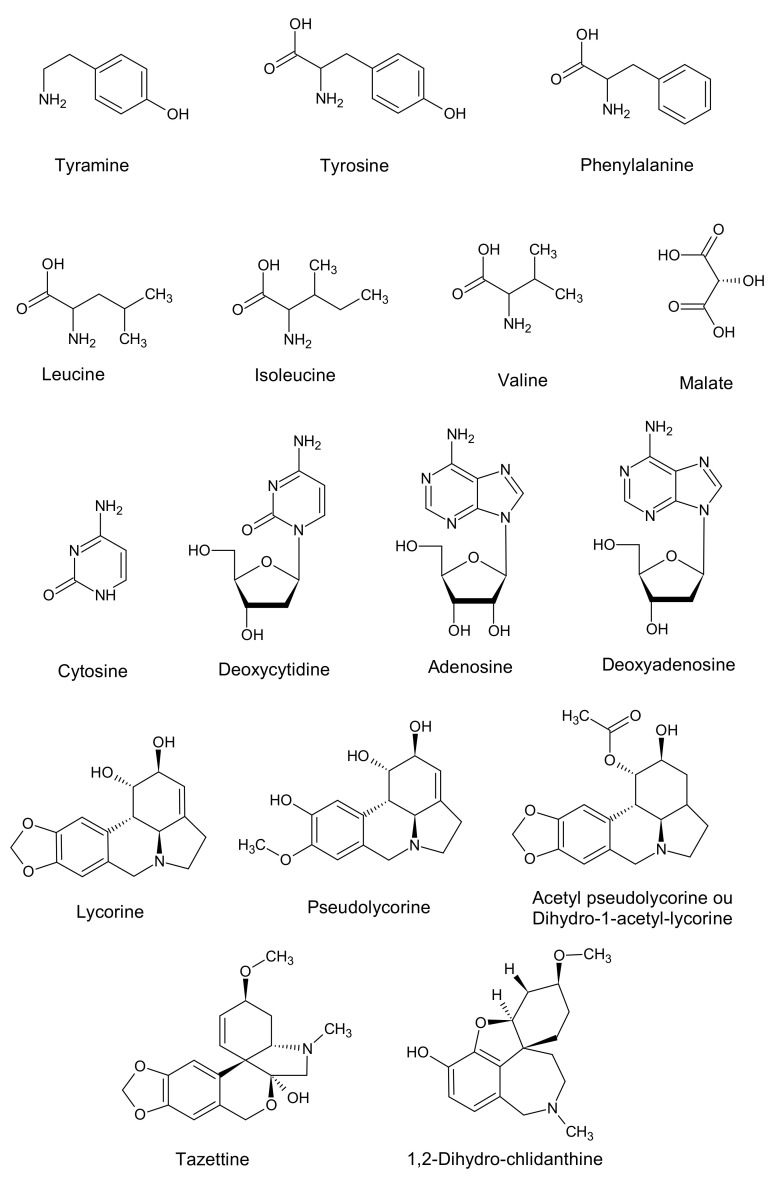
Chemical structures of characteristic compounds found in *Bacillus* sp. endophytic bacterium extract involved in NMR, LC-MS and GC-MS metabolomics analysis.

By metabolic investigation using targeted and untargeted ^1^H NMR-based metabolomics, LC-MS and GC-MS analysis in *Bacillus* sp. endophyte extracts, a total of 11 primary and 6 specialized metabolites were identified. All chemical structures identified in the extract of *Bacillus* sp. are presented in Figure 9. The identification of each of these compounds depended on the method of analysis considered as reported by Tarakemeh et al. [50]. The liquid chromatography coupled with high resolution mass spectrometry (LC-HRMS) was the most sensitive method comparatively to GC-MS and NMR and was able to detect the compounds in amounts lower than 0.02 μg/mL.

The obtained results revealed that the *Bacillus* sp. endophytic bacterium was a potential source of lycorine, an Amaryllidaceae alkaloid of interest.

It is to be noted that NMR metabolomic studies had not been previously used to describe the relation between bulbs, bulblets and endophytes in *L. aestivum* plants. The combination of NMR-based metabolomics and mass spectrometry is a very useful tool for describing the metabolism of Amarylidaceae plants and other biological systems [26,28]

However, large-scale production of lycorine by cultivating this promising endophytic bacterium strain and the investigation of enzymatic systems present in endophytes are in our future projects.

## 3. Materials and Methods

### 3.1. Chemicals

Standards (gallic acid, lycorine, galanthamine, tyramine, tyrosine, α-glucose, β-glucose, sucrose, alanine, valine) are purchased by Sigma Aldrich (Sigma-Aldrich, St. Quentin Fallavier, France). All solvents used are analytical profile grade, deuterated methanol (CD_3_OD), 3-(trimethylsilyl)-1-propanesulfonic acid sodium salt (DSS sodium salt), sodium deuteroxide solution (NaOD) 40 wt.% in deuterium oxide (D_2_O), 99.5 atom % D, ammonium hydroxide solution (NH_4_OH) containing ≥25% NH_3_ basis, were purchased from Sigma Aldrich (Sigma-Aldrich, St. Quentin Fallavier, France). All chemicals for in vitro culture are listed in the article Saliba et al. [29]. SCX cation exchanger column are purchased by Interchim^®^ (Interchim, Montluçon, France).

### 3.2. Plant Materials, Preparation of In Vitro Culture of L. aestivum

*L. aestivum* bulbs were purchased from a local French market (Graines Baumax, Mazirot, France) and voucher specimens were deposited at the herbarium of the Botanic Garden Jean-Marie Pelt (N° 911371) (Nancy, France).

Under sterilized conditions in the laminar air flow, the leaves isolated from the bulbs of this Amaryllidaceae plant were surface sterilized, and initial explants were cultivated on a Murashige and Skoog (MS) culture medium [58] as previously reported by Ptak et al. [59]. For somatic embryo induction, the medium was supplemented with 0.5 µM benzyladenine (BA) combined with 5 µM 4-amino-3,5,6-trichloro-2-pyridinecarboxylic acid (picloram) in accordance to previous results [60,61]. In addition, 3% *w/v* sucrose and 0.8 % *w/v* agar (purified agar from Difco) were added to the medium. All supplements were added before autoclaving. The pH was adjusted to 5.5. Bulblets were regenerated after transferring plants obtained from somatic embryos successively to the MS solid medium containing 5 µM zeatin during 16 weeks, then to the MS solid medium without growth regulators and supplemented with 6% *w/v* sucrose [31]. Cultures were placed in a growth chamber and maintained at 25 ± 2 °C under a 16/8-h (light/dark) photoperiod (Tungsram lamp, 40 WF, 90 µmol m^−2^ s^−1^), and subcultured once every 4 weeks under the same conditions.

### 3.3. Isolation of Endophytic Bacteria

In vitro bulblets of *L. aestivum* were used for the isolation of endophytes. Thin slices about 3–5 mm in length, were obtained from in vitro bulblets. All these explants were placed on nutrient agar (NA) in Petri dishes and incubated in the dark at 28 °C. Growth was monitored periodically for 5 days. Distinct colonies were selected and subcultured on NA to obtain pure isolates. Pure bacterial isolates were preserved in 50% glycerol in a ratio of 500 μL glycerol: 500 μL overnight broth culture and kept at −80 °C.

### 3.4. Identification of Endophytic Bacteria and PCR Amplification

Pure colonies were subjected to Gram staining to establish morphological characteristics such as Gram-stain reaction using a compound bright-field microscope (OLYMPUS CH20BIMF200, Rungis, France) with 100× magnification.

The 16S rDNA genes were amplified from single colonies of isolated strains in a final volume of 25 mL containing 2.5 mL of *Taq* Polymerase buffer 10× (MP Biomedicals, Illkirch-Graffenstaden, France), 200 mM of each dNTP, 1.5 mM magnesium chloride (MgCl_2_), 0.5 mM of each universal primer 27f (50-GAGAGT TTG ATC CTG GCT CAG-30, positions 8–27 of *Escherichia coli* 16S rDNA) and 1492r (50-CTA CGG CTA CCT TGT TAC GA-30, positions 1492–1513 of *E. coli* 16S rDNA) [62], and 0.625 U of *Taq* Polymerase (MP Biomedicals, Illkirch-Graffenstaden, France). DNA amplification was carried out in a thermocycler using the following conditions: 4 min at 94 °C, 35 cycles of 1 min at 94 °C, 1 min at 55 °C, and 2 min at 72 °C, plus an additional 15-min cycle at 72 °C.

### 3.5. In Silico Analysis

Using the BLASTx tool, the sequences were compared to a database available on NCBI containing sequences of RNA16S from bacteria (https://blast.ncbi.nlm.nih.gov/Blast.cgi, accessed on 17 July 2020). The top 100 hits were aligned with Clustal X and the resulting alignment was used to create a phylogenetic tree with MEGA-X. The evolutionary history was inferred by using the Maximum Likelihood method and Tamura-Nei model [63]. Evolutionary analyses were conducted in MEGA X [64].

### 3.6. Preparation of Plants Samples and Endophytic Bacteria for Untargeted NMR-Based Metabolomics

The procedure for obtaining plants samples used in ^1^H NMR based metabolomics entails in vivo bulbs and in vitro bulblets of *L. aestivum* being catted in small pieces, frozen at −80 °C overnight and then lyophilized. The dry materials were blended to obtain a finer powder.

For the endophytic bacterium, a preculture in 2 mL of an NA medium was performed at 32 °C overnight. Twenty Erlenmeyer flasks containing 50 mL of an NA medium were inoculated with the preculture, shaken at 120 rpm and incubated at 32 °C. After 72 h of cultivation, the cultures were centrifuged at 10 °C for 10 min at 4000 rpm, and the bacterial pellets were recovered. The pellets and the liquid media, were frozen and lyophilized for 48 h.

Fifty mg of each sample (in vivo bulbs, in vitro bulblets and bacterial pellets) were directly extracted with a mixture of 0.75 mL of potassium dihydrogenphosphate (KH_2_PO_4_) buffer in deuterium oxide (D_2_O) at pH 6.0 containing 0.1% of 3-(trimethylsilyl)-1-propanesulfonic acid sodium salt (DSS sodium salt), and 0.75 mL CD_3_OD. The mixture was then placed in a vortex mixer. The extraction was realized in an ultrasonic bath for 30 min of sonication. Each sample was centrifuged 2 times for 10 min at 3400 rpm. We use a closed protocol as described in the article by Kim et al. [65]. All deuterated supernatants from in vivo bulbs and in vitro bulblets of *L. aestivum* were transferred into NMR tubes (5 mm) for measurement of their ^1^H spectra. The extraction and analysis were repeated three times.

### 3.7. Preparation of Enriched Endophytic Bacteria Sample for NMR, LC-MS and GC-MS Analysis

For NMR, LC-MS and GC-MS analysis, 50 mg of dried bacterial pellets were extracted with 3 mL of 60% MeOH for 30 min by sonication in an ultrasonic bath followed by maceration for 2 h. The extract was purified using the solid-phase extraction (SPE) purification protocol as previously described by Saliba et al. [29].

The cartridge was washed with 3 mL of alkalized water (2% ammonium hydroxide NH_4_OH, pH = 8) and then eluted with 15 mL of the mobile phase constituted by methanol, distillated water and formic acid (MeOH–H_2_O–HCOOH, 85:10:5 *v/v*). The obtained eluate was then transferred on a SCX cation exchanger column (preconditioned with 2 mL of 2% formic acid in methanol). The cartridge was then rinsed successively with 2 mL of 2% formic acid in methanol, 3 mL of 2% formic acid in acetonitrile and 3 mL of 2% formic acid in acetonitrile. The alkaloids were finally eluted as free bases with 15 mL of 5% NH_4_OH in acetonitrile. The purified extracts were then concentrated. The extraction and analysis were repeated three times.

The purified extract was evaporated and reconstituted in 250 µL of deuterated MeOH. The extract was transferred into Shigemi NMR tubes matched with methanol (Cortecnet Europe, Voisins-Le-Bretonneux, France) for measurement of its ^1^H spectrum.

Then, after evaporation, the purified extract residue was dissolved in 250 µL of acetonitrile and analyzed by LC-MS and GC-MS. The experiments and analysis were repeated three times.

### 3.8. NMR Equipment and Experimental Conditions

^1^H NMR spectra were recorded on a Bruker Avance III 400 spectrometer (Bruker BioSpin, Rheinstetten, Germany), operating at a frequency of 400.13 M + Hz for 1 h and at a temperature of 26 °C using a BBFO Probe and a Bruker sample changer. NMR analyses were performed on the «Plateforme de RMN de l’Institut Jean Barriol», University of Lorraine, France. For quantification, a pulse sequence with presaturation for water suppression was used to acquire ^1^H NMR spectra from all extracts.

For each sample, 512 scans were acquired with 32,768 data points using a spectral width of 4800 Hz, a relaxation delay of 10 s and a pulse width of 14.2 μs. The relaxation delay was chosen to be largely superior to 5 times the longest T1 in the molecules in order to ensure quantitative measurements. The estimated saturation bandwidth is about 60 Hz, as calculated from the presaturation pulse power level. The coupling constant (*J*) is expressed in Hz.

All spectra were manually phased and baseline corrected using the TOPSPIN 4.0.6 (Bruker Biospin GmbH, Rheinstetten, Germany).

DSS sodium salt (at 0.0 ppm) was used as an internal standard for the chemical shift reference and intensity scaling for all ^1^H-NMR signals, as well as a reference in the quantitation of metabolites. Calibration of the data was carried out using TOPSPIN 4.0.6 software (Bruker Biospin GmbH, Rheinstetten, Germany).

The assignments of metabolites in the NMR spectra were made by comparing the proton chemical shifts with literature [66,67,68,69,70,71] or by a comparison with the spectra of authentic compounds recorded in the same solvent conditions (in-house library).

For the quantification [72,73], Equation (1) was used

mX = mSt × (AX/ASt) × (MwX/MwSt) × (NSt/NX)(1)

mX is the unknown mass of the targeted metabolite; mSt is the mass of the DSS; AX and ASt are the integral areas for the selected signals; MwX and MwSt are the molecular weights of the targeted metabolite and DSS; NX and NSt are the number of protons generating the integral signals.

### 3.9. Softwares and the Statistical Analysis

For the quantification, the analyses were repeated three times, and the data were expressed as a mean ± SD, *p* < 0.01.

For the ^1^H NMR spectra processing, bucketing and data export, the interactive tool NMRProcFlow v1.3 was used [74]. In the downloaded ^1^H NMR spectra (Supplementary Figure S2) the baseline correction, ppm calibration and re-alignment were applied. The NMR spectra were reduced in several buckets using the intelligent bucketing method according to De Meyer et al. [75], in a range from 8.2 to 0.5 ppm, excluding the methanol signals (3.34–3.26 ppm).

The obtained bucket table was used to create a data matrix. Normalization, using the option Constant Sum Normalization, was used.

The data matrix, with columns representing the content of each identified metabolite and the lines of each replicate of the three biological origins tested in the study, was imported to SIMCA software version 16.0.2 (Umetrics, Umeå, Sweden), and the PCA was realized using the Pareto scaling method.

### 3.10. LC-MS and GC-MS Equipments and Analysis

The LC system consisted of a U3000-Dionex apparatus with an injector comprising a 1 µL loop and a UV detector at 280 nm. The analytical column used was an Acclaim mixed-mode HILIC-1 (Thermo Scientific, Bellefonte, PA, USA) ID 2.1 mm column (150 mm × 5 µm × 120 Å) and eluted at a flow rate of 200 µL/min using a gradient ranging from 2% solvent B to 25% solvent B in a time span of 26 min and 30 s. Solvent A consisted of pure methanol and solvent B consisted of 10 mM ammonium formate in water at pH 6.8. The Electrospray Ionization—High Resolution Mass Spectrometry (ESI-HRMS) was a micrOTOF_Q_^TM^ apparatus (Bruker Daltonics, Bruker, Bremen, Germany).

The GC system consisted to QP2010-Shimadzu equipment (Shimadzu, Kyoto, Japan) operating in the EI mode at 70 eV. An SLB5 column DB-5 ms (30 m, 0.25 mm film thickness) was employed with a 36 min temperature program of 60–320 at 10 °C/min followed by a 10 min hold at 320 °C. The injector temperature was 250 °C; the flow rate of the carrier gas (helium) was 1 mL/min; and the split ratio was 1:50. The interval of the scan *m/z* was between 35 and 900 and the identification of the compounds was based on an individual spectrum comparison of each compound in the Shimadzu NIST08 data. The retention index was calculated using the mixture of alkanes standard from C10 to C40 (Merck KGaA, Darmstadt, Germany).

## 4. Conclusions

For the first time, endophytic bacteria were isolated from *Leucojum aestivum* in in vitro bulblets. To date, no other studies have investigated the presence of endophytic bacteria in *Leucojum* species (neither bulbs nor bulblets). The discovery of endophytic bacteria belonging to the *Bacillus* genus living in tissue cultures of this plant and their capacity to synthesize Amaryllidaceae alkaloids opens new prospects for producing these added-value specialized metabolites, which are accumulated at low levels in plants. The coupling between NMR-based metabolomics and mass spectrometry combined with multivariate statistical analysis was a reliable method for comparing the metabolism of *L. aestivum* and its endophytes. For several years, researchers tried to enhance the biosynthesis of Amaryllidaceae-specialized metabolites, especially galanthamine and lycorine production via in vitro cultures (a precursor addition to the culture medium for in vitro culture conditions modulation and optimization, bioreactors RITA^®^)^.^ These techniques showed interesting results but neither offered an economically viable protocol for an effective scale-up due to the complexity of managing plant bioreactors and the relatively high costs of such technologies. Bacterial cultures can be scaled-up because culturing conditions, specialized metabolite extraction, culturing costs and time scales offer an economically viable biotechnology.

## Figures and Tables

**Table 1:** Identified molecules by metabolic fingerprinting using ^1^H NMR spectra in in vivo bulbs, in vitro bulblets of *L. aestivum* and endophytes *Bacillus* sp.

Molecules	Molecular Weight	Molecular Formula	Chemical Shifts, Multiplicity (ppm), *J* (Hz)	Extract from In Vivo *L. aestivum* Bulb	Extract from In Vitro *L. aestivum* Bulblet	Extract from *Bacillus* sp.
Adenine	135,113	C_5_H_5_N_5_	8.2 (s), 8.21 (s)	−	−	Y
Cytosine	111.1	C_4_H_5_N_3_O	7.52 (s), 7.51 (s)	−	−	Y
Phenylalanine	165.19	C_9_H_11_NO_2_	7.42–7.33 (m), 3.09 (dd, *J* = 14.8, 8.3)	−	−	Y
Tyrosine	181.19	C_9_H_11_NO_3_	7.16 (d, *J* = 8.4), 6.83 (d, *J* = 8.5)	Y	Y	Y
Tyramine	137.18	C_8_H_11_NO	7.11 (d, *J* = 8.6) 6.71,(d, *J* = 8.5)	Y	Y	Y
Gallic acid	170.12	C_7_H_6_O_5_	7.02 (s)	Y	Y	−
Lycorine	287.32	C_16_H_17_NO_4_	7.00 (s), 6.85 (s)	Y	Y	−
Galanthamine	287.35	C_17_H_21_NO_3_	6.95 (d, *J* = 8.5) 6.89 (d, *J* = 8.5)	Y	−	−
Sucrose	342.30	C_12_H_22_O_11_	5.39 (d, *J* = 3.8)	Y	Y	−
α-Glucose	180.16	C_6_H_12_O_6_	5.17 (d, *J* = 3.8)	Y	Y	−
β-Glucose	180.16	C_6_H_12_O_6_	4.57 (d, *J* = 7.9)	Y	Y	−
Malate	134.09	C_4_H_6_O_5_	4.34 (dd, *J* = 6.6 Hz, 4.7 Hz)	−	−	Y
Oxaloacetic acid	132.07	C_4_H_4_O_5_	3.64 (s)	Y	Y	−
Asparagine	132.11	C_4_H_8_N_2_O_3_	3.94 (dd, *J* = 8.0, 4.0)	Y	Y	−
Aspartate	133.10	C_4_H_7_NO_4_	2.78 (dd, *J* = 1.7, 5.3)	Y	Y	−
Citrate	192.12	C_6_H_8_O_7_	2.74 (d, *J* = 17.6 Hz),	−	Y	−
Alanine	89.09	C_3_H_7_NO_2_	1.46 (d, *J* = 7.2)	Y	Y	−
Threonine	119.12	C_4_H_9_NO_3_	1.33 (d, *J* = 6.6)	Y	Y	−
Valine	117.15	C_5_H_11_NO_2_	1.00 (d, *J* = 6.8) 1.05 (d, *J* = 6.8)	Y	Y	Y
Isoleucine	131.17	C_6_H_13_NO_2_	0.96 (t, *J* = 7.4) 1.03 (d, *J* = 6.8)	Y	Y	Y
Leucine	131.17	C_6_H_13_NO_2_	1.05 (d, *J* = 7) 1.01 (t, *J* = 7)	−	−	Y
Fatty acids (as oleic acid)	282.5	C_18_H_34_O_2_	0.89 (d, *J* = 7.1)	Y	Y	Y

400 M + Hz, DSS = 3-(trimethylsilyl)-1-propanesulfonic acid sodium salt is used as chemical shift reference. The symbol (−) means that compound was not observed, the symbol (Y) means the presence of compound.

**Table 2:** Amaryllidaceae alkaloids and other compounds identified in endophytic bacteria of in vitro bulblets of *L. aestivum* by LC-MS using positive ionization mode.

Compounds Name	Retention Time (min)	Molecular Formula	Molecular Mass [M+H]^+^	Identification Methods
Methyl-phenylalanine	1.9	C_10_H_13_NO_2_	180.1025	b, d, e, f
**Tazettine**	3.2	C_18_H_21_NO_5_	332.1486	c, e, f
Deoxyadenosine	4.6	C_10_H_13_N_5_O_3_	252.1097	c, d, e
Adenosine	4.9	C_10_H_13_N_5_O_4_	268.10481	b, c, d, e
**Lycorine**	6.1	C_16_H_17_NO_4_	288.1231	a, c, e, f
Deoxycytidine	6.4	C_9_H_13_N_3_O4	228.0997	c, d, e
**Pseudolycorine**	8	C_16_H_19_NO4	290.14	e, f
**Acetyl pseudolycorine**	11.5	C_18_H_21_NO_5_	332.1498	c, e

(a) original standard, (b) GNPS [46], (c) Metlin [47], (d) Massbank [48], (e) Sirius [49], (f) manualy or house database (DataNat). In black bold are presented Amaryllidaceae alkaloids.

**Table 3:** Amaryllidaceae alkaloids identified by GC-MS in purified endophytic *Bacillus* sp. extract.

Compound Name	Retention Time (min)	Molecular Mass	Molecular Formula	Retention Index
Tazettine	12.84	331	C_18_H_21_NO_5_	2415
1,2-Dihydro-chlidanthine	15.15	289	C_17_H_23_NO_3_	2211

## Data Availability

Not applicable.

## Supplementary Materials

Supplementary Materials can be found at https://www.mdpi.com/1422-0067/22/4/1773/s1. Insertion S1: Bacteria strains compared to isolated strain (*Bacillus* sp.) from *L. aestivum* in vitro bulblet; Figure S1: MSMS of lycorine derived from endophytic *Bacillus* sp. isolated from *L. aestivum* in vitro bulblet; Figure S2: ^1^H NMR Spectra in NMRProcFlow.

## Glossary

MeOH	Methanol
CH_3_CN	Acetonitrile
HCOOH	Formic acid
NH_4_OH	Ammonium hydroxide
LA	*Leucojum aestivum*
MS	Murashige and Skoog
NA	Nutrient agar
DW	Dry weight
SPE	Solid phase extraction
NMR	Nuclear Magnetic Resonance
ESI-HRMS	Electrospray Ionization—High Resolution Mass Spectrometry
MS	Mass spectrometry
LC	Liquid chromatography
GC	Gas chromatography
