# Supplementary material for: Molecular Identification of Endophytic Bacteria in Leucojum aestivum In Vitro Culture, NMR-Based Metabolomics Study and LC-MS Analysis Leading to Potential Amaryllidaceae Alkaloid Production
Source: Int J Mol Sci. 2021 Feb 10;22(4):1773. doi: 10.3390/ijms22041773 (PMC7916811; doi:10.3390/ijms22041773)
Supplement: Supplementary file 1 [file ijms-22-01773-s001.pdf]

Supplementary materials

Insertion S1: Bacteria strains compared to isolated strain (Bacillus sp.) from L. aestivum in vitro bulblet.

The isolated strain from L. aestivum in vitro bulblet (highlighted by an orange arrow in the Figure 2 of the manuscript), has been compared to: R_115334.1_Bacillus_pumilus_strain_CIP_52.67,NR_043242.1_Bacillus_pumilus_strain_ATCC_7061,NR_148786.1_Bacillus_zhangzhouensis_strain_MCCC_1A08372,NR_112637.1_Bacillus_pumilus_strain_NBRC_12092,NR_113945.1_Bacillus_safensis_strain_NBRC_100820,NR_041794.1_Bacillus_safensis_FOZ6b,NR_148787.1_Bacillus_australimaris_strain_MCCC_1A05787,NR_118441.1_Bacillus_stratosphericus_strain_41KF2a,NR_042337.1_Bacillus_altitudinis_41KF2b,NR_042339.1_Bacillus_aerophilus_strain_28K,NR_042336.1_Bacillus_stratosphericus_strain_41KF2a,NR_118439.1_Bacillus_aerius_strain_24K,NR_148244.1_Bacillus_xiamenensis_strain_MCCC_1A00008,NR_118381.1_Bacillus_pumilus_strain_SBMP2,NR_043268.1_Bacillus_idriensis_strain_SMC_43522,NR_116191.1_Bacillus_pumilus_strain_NRRL_NRS272,NR_042538.1_Salirhabdus_euzebyi_strain_CVS14,NR_116287.1_Falsibacillus_pallidus_strain_CW_7,NR_115578.1_Bacillus_benzoevorans_strain_DSM_5391,NR_157636.1_Bacillus_mangrovi_strain_AK61,NR_102783.2_Bacillus_subtilis_subsp._subtilis_strain_168,NR_024696.1_Bacillus_vallismortis_strain_DSM_11031,NR_116022.1_Bacillus_amyloliquefaciens_strain_BCRC_11601,NR_116240.1_Bacillus_velezensis_strain_CBMB205,NR_024817.1_Bacillus_asahii_strain_MA001,NR_043334.1_Bacillus_niabensis_strain_4T19,NR_125634.1_Salinibacillus_xinjiangensis_strain_J4,NR_112685.1_Bacillus_amyloliquefaciens_strain_NBRC_15535,NR_113994.1_Bacillus_vallismortis_strain_NBRC_101236,NR_024689.1_Bacillus_atrophaeus_strain_JCM_9070,NR_146005.1_Bacillus_malikii_strain_NCCP662,NR_151979.1_Bacillus_oryzisoli_strain_1DS310,NR_112723.1_Bacillus_atrophaeus_strain_NBRC_15539,NR_117946.1_Bacillus_amyloliquefaciens_strain_MPA_1034,NR_041455.1_Bacillus_amyloliquefaciens_strain_NBRC_15535,NR_104919.1_Bacillus_tequilensis_strain_10b,NR_117274.1_Bacillus_siamensis_KCTC_13613_strain_PDA10,NR_157742.1_Bacillus_wudalianchiensis_strain_FJAT27215,NR_042974.1_Bacillus_indicus_strain_JG30,NR_042286.1_Bacillus_herbersteinensis_strain_D1,5,NR_044828.1_Bacillus_benzoevorans_strain_NCIMB_12555,NR_029022.1_Bacillus_indicus_strain_Sd/3,NR_118972.1_Bacillus_subtilis_strain_NCDO_1769,NR_027552.1_Bacillus_subtilis_strain_DSM_10,NR_075005.2_Bacillus_velezensis_strain_FZB42,NR_112116.2_Bacillus_subtilis_strain_IAM_12118,NR_159904.1_Bacillus_lacus_strain_AK74,NR_024931.1_Bacillus_subtilis_subsp._spizizenii_strain_NRRL_B23049,NR_151897.1_Bacillus_nakamurai_strain_NRRL_B41091,NR_116017.1_Bacillus_subtilis_strain_BCRC_10255,NR_115063.1_Bacillus_halotolerans_strain_DSM_8802,NR_113265.1_Bacillus_subtilis_strain_JCM_1465,NR_115282.1_Bacillus_halotolerans_strain_CR95,NR_112686.1_Bacillus_subtilis_subsp._spizizenii_strain_NBRC_101239,NR_112629.1_Bacillus_subtilis_strain_NBRC_13719,NR_115931.1_Bacillus_halotolerans_strain_LMG_22477,NR_115930.1_Bacillus_halotolerans_strain_CECT_5687,NR_115929.1_Bacillus_halotolerans_strain_LMG_22476,NR_118290.1_Bacillus_mojavensis_strain_ifo_15718,NR_118456.1_Quasibacillus_thermotolerans_strain_SgZ8,NR_043015.1_Bacillus_litoralis_strain_SW211,NR_118950.1_Bacillus_amyloliquefaciens_DSM_7_strain_ATCC_23350,NR_157608.1_Bacillus_swezeyi_strain_NRRL_B41294,NR_024693.1_Bacillus_mojavensis_strain_IFO15718,NR_115325.1_Bacillus_nematocida_strain_B16,NR_104873.1_Bacillus_subtilis_subsp._inaquosorum_strain_BGSC_3A28,NR_112725.1_Bacillus_mojavensis_strain_NBRC_15718,NR_159903.1_Ornithinibacillus_salinisoli_strain_LCB256,NR_159145.1_Salirhabdus_salicampi_strain_BH128,NR_132682.1_Bacillus_kyonggiensis_strain_NB22,NR_044532.1_Pontibacill

us_halophilus_strain_JSM_076056,NR_112632.1_Bacillus_circulans_strain_NBRC_13626,NR_043084.1_Bacillus_koreensis_strain_BR030,NR_041379.1_Bacillus_panaciterrae_strain_Gsoil_1517,NR_158107.1_Bacillus_endozanthoxylicus_strain_1404,NR_109140.1_Bacillus_persicus_strain_B48,NR_104566.1_Bacillus_circulans_strain_ATCC_4513,NR_117285.1_Bacillus_oceanisediminis_strain_H2,NR_029057.1_Macrococcus_lamae_strain_R_16089,NR_149779.1_Bacillus_loiseleuriae_strain_FJAT27997, NR_146034.1_Bacillus_depressus_strain_BZ1,NR_115877.1_Bacillus_canaveralius_strain_KSC_SF8b,NR_133974.1_Bacillus_huizhouensis_strain_GSS03,NR_125629.1_Bacillus_massilioanorexius_AP8, NR_109671.1_Bacillus_abyssalis_strain_SCSIO_15042,NR_036847.1_Macrococcus_brunensis_strain_CCM_4811,NR_041641.1_Bacillus_azotoformans_LMG_9581,NR_113993.1_Bacillus_sonorensis_strain_NBRC_101234,NR_043700.1_Oceanobacillus_chironomi_strain_T3944D,NR_108491.1_Bacillus_gottheilii_strain_WCC_4585,NR_147397.1_Bacillus_marasmi_strain_MarseilleP3556,NR_145585.1_Bacillus_gossypii_strain_JM267,NR_156041.1_Bacillus_maritimus_strain_KS169,NR_148614.1_Bacillus_ectoiniformans_strain_NE14,NR_125726.1_Jeotgalibacillus_soli_strain_P9,>NR_117927.1_Ornithinibacillus_scapharcae_TW25,NR_148280.1_Bacillus_cihuensis_strain_FJAT14515,NR_043267.1_Bacillus_infantis_strain_SMC_43521,NR_109068.1_Bacillus_ginsengisoli_strain_DCY53,NR_028709.1_Bacillus_siralis_strain_171544 The evolutionary history was inferred by using the Maximum Likelihood method and Tamura-Nei model [1]. Evolutionary analyses were conducted in MEGA X [2].

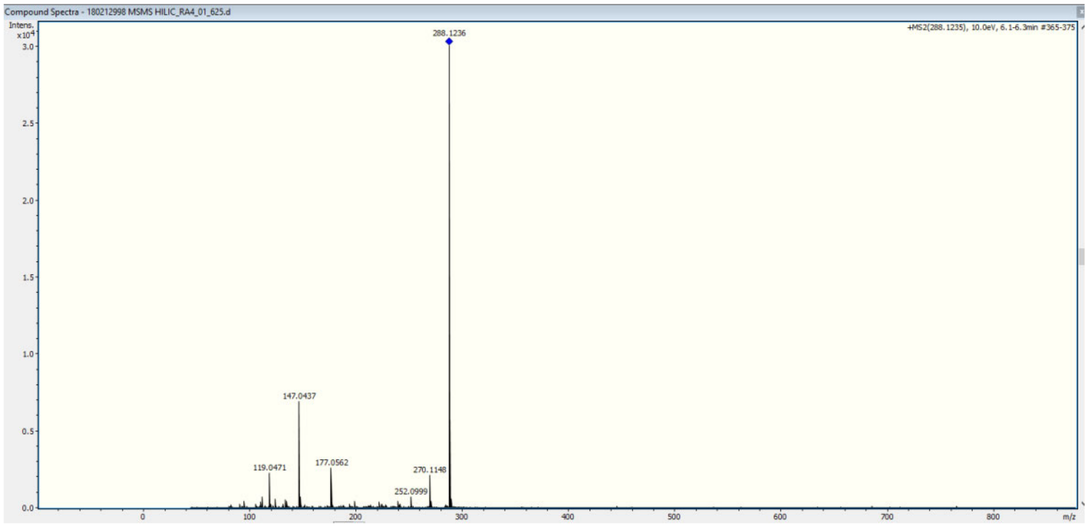

Figure S1. MSMS of lycorine derived from endophytic *Bacillus* sp. isolated from *L. aestivum* in vitro bulblet.

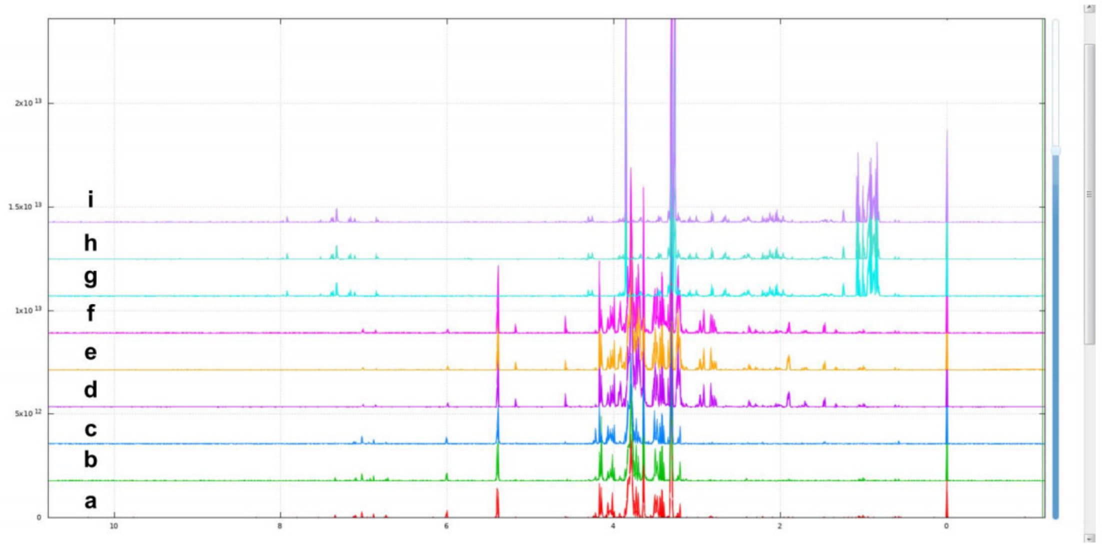

Figure S2. 1H NMR Spectra in NMRProcFlow. *Leucojum aestivum* in vivo bulb (spectra a, b, c); Leucojum aestivum in vitro bulblets (spectra d, e, f), *Bacillus* sp. (spectra g,h, i).

References

1. Tamura, K.; Nei, M. Estimation of the number of nucleotide substitutions in the control region of mitochondrial DNA in humans and chimpanzees. *Mol. Biol. Evol.* **1993**, *10*, 512–526, doi:10.1093/oxfordjournals.molbev.a040023.
2. Kumar, S.; Stecher, G.; Li, M.; Knyaz, C.; Tamura, K. MEGA X: Molecular Evolutionary Genetics Analysis across Computing Platforms. *Mol. Biol. Evol.* **2018**, *35*, 1547–1549, doi:10.1093/molbev/msy096.
